# PGC-1α-Mediated Branched-Chain Amino Acid Metabolism in the Skeletal Muscle

**DOI:** 10.1371/journal.pone.0091006

**Published:** 2014-03-17

**Authors:** Yukino Hatazawa, Miki Tadaishi, Yuta Nagaike, Akihito Morita, Yoshihiro Ogawa, Osamu Ezaki, Takako Takai-Igarashi, Yasuyuki Kitaura, Yoshiharu Shimomura, Yasutomi Kamei, Shinji Miura

**Affiliations:** 1 Department of Molecular Endocrinology and Metabolism, Graduate School of Medical and Dental Sciences, Tokyo Medical and Dental University, Tokyo, Japan; 2 Laboratory of Molecular Nutrition, Graduate School of Environmental and Life Science, Kyoto Prefectural University, Kyoto, Japan; 3 Department of Food Function and Labeling, National Institute of Health and Nutrition, Tokyo, Japan; 4 Laboratory of Nutritional Biochemistry, Graduate School of Nutritional and Environmental Sciences, University of Shizuoka, Shizuoka, Japan; 5 Department of Human Health and Design, Showa Women's University, Tokyo, Japan; 6 Department of Health Record Informatics, Tohoku Medical Megabank Organization, Tohoku University, Miyagi, Japan; 7 Department of Applied Molecular Biosciences, Graduate School of Bioagricultural Sciences, Nagoya University, Nagoya, Japan; University of Debrecen, Hungary

## Abstract

Peroxisome proliferator-activated receptor (PPAR) γ coactivator 1α (PGC-1α) is a coactivator of various nuclear receptors and other transcription factors, which is involved in the regulation of energy metabolism, thermogenesis, and other biological processes that control phenotypic characteristics of various organ systems including skeletal muscle. PGC-1α in skeletal muscle is considered to be involved in contractile protein function, mitochondrial function, metabolic regulation, intracellular signaling, and transcriptional responses. Branched-chain amino acid (BCAA) metabolism mainly occurs in skeletal muscle mitochondria, and enzymes related to BCAA metabolism are increased by exercise. Using murine skeletal muscle overexpressing PGC-1α and cultured cells, we investigated whether PGC-1α stimulates BCAA metabolism by increasing the expression of enzymes involved in BCAA metabolism. Transgenic mice overexpressing PGC-1α specifically in the skeletal muscle had increased the expression of branched-chain aminotransferase (BCAT) 2, branched-chain α-keto acid dehydrogenase (BCKDH), which catabolize BCAA. The expression of BCKDH kinase (BCKDK), which phosphorylates BCKDH and suppresses its enzymatic activity, was unchanged. The amount of BCAA in the skeletal muscle was significantly decreased in the transgenic mice compared with that in the wild-type mice. The amount of glutamic acid, a metabolite of BCAA catabolism, was increased in the transgenic mice, suggesting the activation of muscle BCAA metabolism by PGC-1α. In C2C12 cells, the overexpression of PGC-1α significantly increased the expression of BCAT2 and BCKDH but not BCKDK. Thus, PGC-1α in the skeletal muscle is considered to significantly contribute to BCAA metabolism.

## Introduction

Peroxisome proliferator-activated receptor (PPAR) γ coactivator 1 α (PGC-1α) was identified as a nuclear receptor coactivator of PPARγ in brown adipose tissue and found to be upregulated in brown adipose tissue and skeletal muscle in response to cold exposure [1]. PGC-1α is now known to be involved not only in the regulation of thermogenesis but also in energy metabolism and other biological processes that are critical in controlling phenotypic characteristics of various organ systems [1]–[5]. PGC-1α coactivates a broad range of transcription factors, including PPARs, glucocorticoid receptor (GR), nuclear respiratory factors, myocyte enhancing factors, estrogen-related receptor, and forkhead box O1 [6]–[9]. PGC-1α acts through the recruitment of coactivators with histone acetyl transferase activity as well as interaction with proteins involved in transcriptional initiation and RNA processing [10].

It has recently been shown that there are several isoforms of PGC-1α mRNA [11]–[14]. We previously reported that among the PGC-1α isoforms, PGC-1α-b expression was markedly increased in response to exercise [15]. PGC-1α-b, considered to be similar in function to PGC-1α1 (originally found full-length PGC-1α [1]), structurally differs by 16 amino acids at its amino terminal [12]. We demonstrated that overexpression of PGC-1α-b in skeletal muscle but not in heart increases mitochondrial biogenesis and capillary density, contributing to improved exercise capacity [4]. Moreover, animal and cellular genetic models with altered expression of the PGC-1α gene have much evidence for the role of PGC-1α in fiber type specificity [16], [17], mitochondrial biogenesis [17]–[19], angiogenesis [20], and improved exercise performance [21].

Mammalian cells have a high capacity system for oxidative disposal of branched-chain amino acids (BCAA). In contrast to other essential amino acids, which are primarily oxidized in the liver, the most active system for the oxidation of BCAA is located in skeletal muscle cells [22]. The degradation of BCAA mainly occurs in the mitochondria via reversible transamination by branched-chain aminotransferase (BCAT) to produce the corresponding branched-chain α-keto acids (BCKA), which in turn are subjected to oxidative decarboxylation by branched-chain α-keto acid dehydrogenase (BCKDH) to produce CoA esters. The enzymes that catalyze these two reactions are common to the three BCAA (Val, Leu, and Ile). The second step enzyme, BCKDH, catalyzes an irreversible reaction that commits individual BCKA to their respective degradation pathways [23] and is considered to be the most important regulatory enzyme in the catabolism of the three BCAA [24]. BCKDH activity is regulated by BCKDH kinase (BCKDK); BCKDH phosphorylation attenuates its enzyme activity [23]. In this study, microarray analysis revealed that the BCAA catabolic pathway was coordinately activated in skeletal muscle of transgenic mice overexpressing PGC-1α. Thus, we investigated whether PGC-1α stimulates BCAA metabolism with an increase in the expression of enzymes involved in BCAA metabolism, such as BCAT, BCKDH and BCKDK, using cultured cells and murine skeletal muscle overexpressing PGC-1α.

## Methods

### Transgenic (Tg) mice

Tg mice overexpressing PGC-1α-b in skeletal muscle (hereafter, PGC-1α Tg mice or just Tg mice) were generated as described [12]. In brief, the human α-skeletal actin promoter was used to express PGC-1α-b in skeletal muscle (C57BL6 background). We used the B line of Tg mice in this study; Two independent lines of Tg mice showed similar phenotypes in our previous study [4]. Mice were killed by rapid neck disarticulation. A total of 32 mice were used.

### Ethics Statement

Mice were cared for in accordance with the National Institutes of Health (NIH) Guide for the Care and Use of Laboratory Animals and our institutional guidelines. All animal experiments were performed with the approval of the National Institute of Health and Nutrition Ethics Committee on Animal Research (approval ID: No 908, 1008, and 1111) and Institutional Animal Care and Use Committee of University of Shizuoka.

### cDNA microarray analysis

RNA was isolated from skeletal muscle (gastrocnemius) of Tg mice (age, 12 weeks) and age-matched WT control mice. Samples from WT and Tg mice (N = 5) were pooled and used. Each sample was labeled with a cyanine 3-CTP using the Low Input Quick Amp Labeling Kit (Agilent Technologies, Inc., Santa Clara, CA) and hybridized to the Agilent whole mouse genome microarray (4×44K), which contains 41,534 genes including expressed sequence tags. Signal detection and data analysis were performed according to the manufacturer's instructions [25].

### Functional annotation analysis in genes up-regulated by PGC-1α overexpression

We conducted pathway analysis using the Kyoto Encyclopedia of Genes and Genomes (KEGG) database resource with DAVID v6.7 [26], which is a web application providing a comprehensive set of functional annotation tools to understand the biological meaning of a large list of genes. A list of gene symbols that showed increased expression in skeletal muscle of PGC-1α Tg mice was submitted, and a significant overrepresentation of the KEGG pathway was detected.

### Bioinformatics analysis of transcription factors enriched in the BCAA metabolic pathway genes up-regulated in PGC-1α Tg mice

We employed ChIP Enrichment analysis (ChEA) software [27] to explore the transcription factors involved in the regulation of genes whose expression was induced by PGC-1α overexpression classified as BCAA metabolic pathway by DAVID v6.7 [26]. ChEA is a tool that computes over-representation of transcription factor targets from the database of ChIP-seq and ChIP-chip experiments [27]. The database as of Dec 23, 2013 contains 471,284 extracted entries, from 228 publications, describing the binding of 203 transcription factors to 47,119 target genes (http://amp.pham.mssm.edu/lib/chea.jsp).

### Quantitative real-time RT-PCR analysis

Total RNA was prepared usnig TRIzol (Life Technologies Japan, Tokyo, Japan). cDNA was synthesized from 1 μg of total RNA using the QuantiTect Rev. Transcription Kit (QIAGEN K.K, Tokyo, Japan). Gene expression levels were measured as described [25]. The following primers were used:

BCAT2 Fw, 5′-CGGACCCTTCATTCGTCAGA -3′; BCAT2 Rv, 5′-CCATAGTTCCCCCCCAACTT-3′; BCKDHa Fw, 5′-CCAGGGTTGGTGGGATGAG-3′; BCKDHa Rv, 5′-GGCTTCCATGACCTTCTTTCG-3′; BCKDK Fw, 5′-GATCCGAATGCTGGCTACTCA-3′; BCKDK Rv, 5′-GCCAACAAAATCAGGCTTGTC-3′; PGC-1α Fw, 5′-CGGAAATCATATCCAACCAG-3′; PGC-1α Rv, 5′-TGAGGACCGCTAGCAAGTTTG-3′; 36B4 Fw, 5′-GGCCCTGCACTCTCGCTTTC-3′; 36B4 Rv, 5′-TGCCAGGACGCGCTTGT -3′;

### Stable cell lines

PlatE cells were cultured in 90-mm dishes and transfected at 70% confluence using Lipofectamine 2000 (Life Technologies Japan, Tokyo, Japan) according to the manufacturer's instructions using 2 μg pMX-derived expression plasmid [28] containing PGC-1α cDNA or vector alone. Virus-containing supernatants were harvested 48 h after transfection and added to dishes of C2C12 cells, which were selected using 5 μg/ml puromycin to eliminate uninfected cells. After drug selection, virally infected stable cells were cultured to confluence in Dulbecco's Modified Eagle Medium (DMEM) containing 10% fetal calf serum, and the medium was changed every 2 days. On 3 day after confluence, cells were used for RNA preparation.

### Western blotting analysis

Frozen skeletal muscle (gastrocnemius) was homogenized in RIPA Lysis Buffer (25 mM Tris-HCl pH 7.6, 150 mM NaCl, 1% NP-40, 1% sodium deoxycholate, 0.1% SDS) containing 0.2 mM sodium orthovanadate, 2 mM phenylmethylsulfonyl fluoride, and protease inhibitor cocktail (1/100 volume) (Sigma Aldrich Japan, K.K. Tokyo, Japan). The supernatant was separated by centrifugation at 20,400 g for 15 min at 4°C. Protein from the supernatant (20 μg) was applied onto an SDS-PAGE gel. A commercially available precast ready-made gel (10% acrylamide, e-PAGEL, ATTO Co. Tokyo, Japan) was used. The following primary antibodies were used for Western blotting: anti-PGC-1α (Rabbit polyclonal IgG) against the carboxyl terminus 777–797 (Millipore, Billerica, MA), and anti-BCKDH (rabbit polyclonal, [29]).

### Amino acid analysis

Skeletal muscle (gastrocnemius) and blood from PGC-1α Tg mice (in the feeding condition) were used for amino acid analysis. In addition, C2C12 cells overexpressing PGC-1α were examined for the amino acid content. Skeletal muscle and C2C12 cells were extracted with methanol/chloroform/water (5/5/2 in volume), and centrifuged. The supernatant was dried with nitrogen gas and dissolved in water. Amino acid levels were measured by HPLC assays (SRL, Tokyo, Japan).

### Statistical analysis

Statistical analysis was performed using Student's t test. Data were expressed as the mean ± SE. P value <0.05 was considered statistically significant. Standard P-values (Fisher's exact test) and Benjamini P-values were evaluated for functional annotation analysis. The Fisher exact test with the Bonferroni's correction P-values were evaluated for transcription factor search analysis.

## Results And Discussion

### Increased BCAA metabolism in skeletal muscle of PGC-1α Tg mice

**Figure 1:**
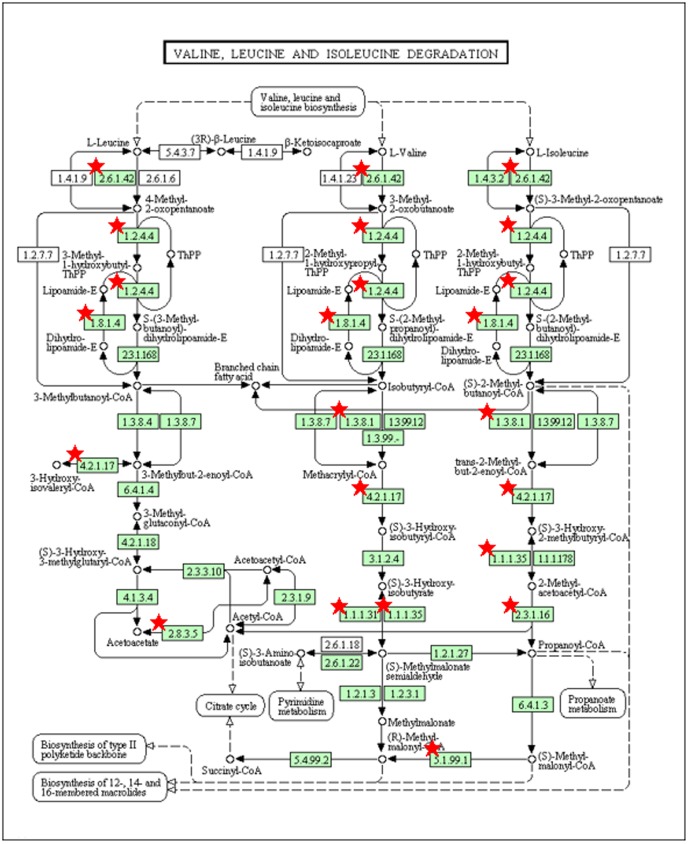
Pathway map of Val, Leu, and Ile degradation. Individual gene name of the KEGG pathway of Val, Leu, and Ile degradation extracted by pathway analysis is shown as a metabolic map. Red asterisks indicate increased gene expression by microarray of Tg mice. Gene names corresponding to enzyme numbers with red asterisks are as follows: 2.8.3.5, 3-oxoacid CoA transferase 1; 2.3.1.16, acetyl-Coenzyme A acyltransferase 2 (mitochondrial 3-oxoacyl-Coenzyme A thiolase); 1.3.8.1, acyl-Coenzyme A dehydrogenase, short chain; 2.6.1.42, branched chain aminotransferase 2, mitochondrial; 1.2.4.4, branched chain ketoacid dehydrogenase E1, alpha polypeptide; 1.8.1.4, dihydrolipoamide dehydrogenase; 1.1.1.35, hydroxyacyl-Coenzyme A dehydrogenase; 4.2.1.17, hydroxyacyl-Coenzyme A dehydrogenase/3-ketoacyl-Coenzyme A thiolase/enoyl-Coenzyme A hydratase (trifunctional protein), alpha subunit; 5.1.99.1, methylmalonyl CoA epimerase; 1.1.1.31, 3-hydroxyisobutyrate dehydrogenase.

To characterize the phenotype of the skeletal muscle of Tg mice, we performed microarray analysis of gene expression. Microarray analysis revealed that the expression of many genes was changed in Tg mice as compared with that in WT mice. Among these, 315 genes (Table S1) were up-regulated (more than 2.5-hold) and used to conduct pathway analysis, which detected 7 categories (Table 1), including oxidative phosphorylation, TCA cycle, and fatty acid metabolism, which were related to mitochondrial function. These results were consistent with previous reports that PGC-1α increases the mitochondrial number and enhances their function [3], [5]. We observed pathway categories of Parkinson's disease, Huntington's disease and Alzheimer's disease (Table 1), and individual genes increased in the categories were all related to mitochondrial functions (data not shown). In addition, we found that BCAA metabolic pathway (i.e., Val, Leu, Ile degradation, Table 1). As shown in Figure 1, gene expression of many enzymes involved in BCAA catabolism was increased, suggesting that PGC-1α stimulates BCAA metabolism in skeletal muscle. Thus, we examined whether PGC-1α stimulates BCAA metabolism.

**Table 1:** Pathway analysis.

Categories of pathway analysis	P-Value	Benjamini
Oxidative phosphorylation	1.10E-13	1.00E-11
Parkinson's disease	1.70E-13	8.10E-12
Citrate cycle (TCA cycle)	3.10E-12	9.70E-11
Huntington's disease	9.10E-12	2.10E-10
Alzheimer's disease	5.30E-10	9.90E-09
Valine, leucine and isoleucine degradation	7.10E-09	1.10E-07
Fatty acid metabolism	9.10E-08	1.20E-06

Compared with WT mice, 315 genes were found to be up-regulated in PGC-1α Tg mice by microarray and classified into KEGG pathway analysis as described in Methods.

### Levels of BCAA and its catabolic enzymes in skeletal muscle of PGC-1α Tg mice

**Figure 2:**
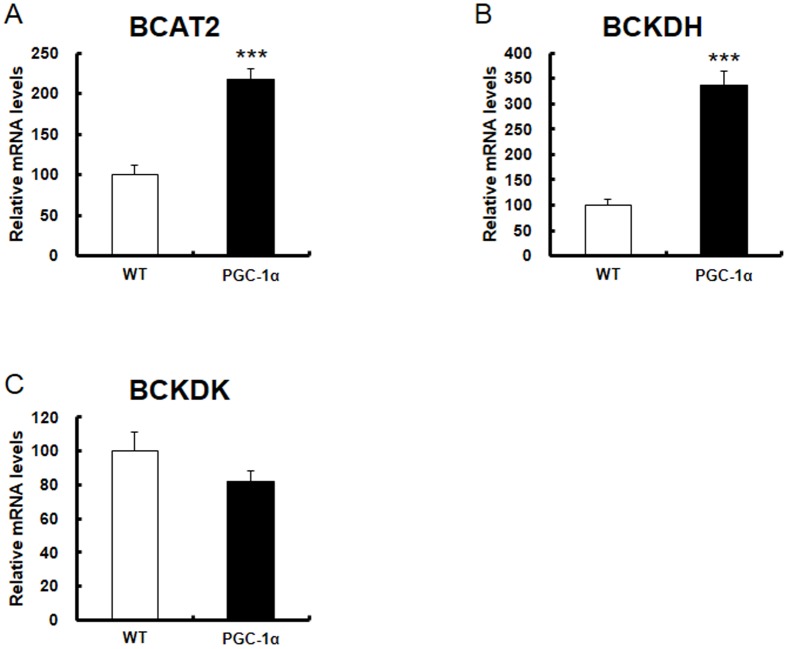
Gene expression of BCAA metabolic enzyme in skeletal muscle of PGC-1α Tg mice. Expression of A) BCAT2, B) BCKDH, and C) BCKDK genes in WT (control; open columns, N = 9) and PGC-1α Tg (filled columns, N = 7) mice by quantitative real-time RT-PCR. RNA was obtained from mice with feeding condition. These samples were as used in [4]. In the sample, PGC-1α expression was 30 fold higher in Tg mice than in WT mice (Fig. 1 of [4]). The relative values are shown (the control is set as 100). ***P<0.001.

**Figure 3:**
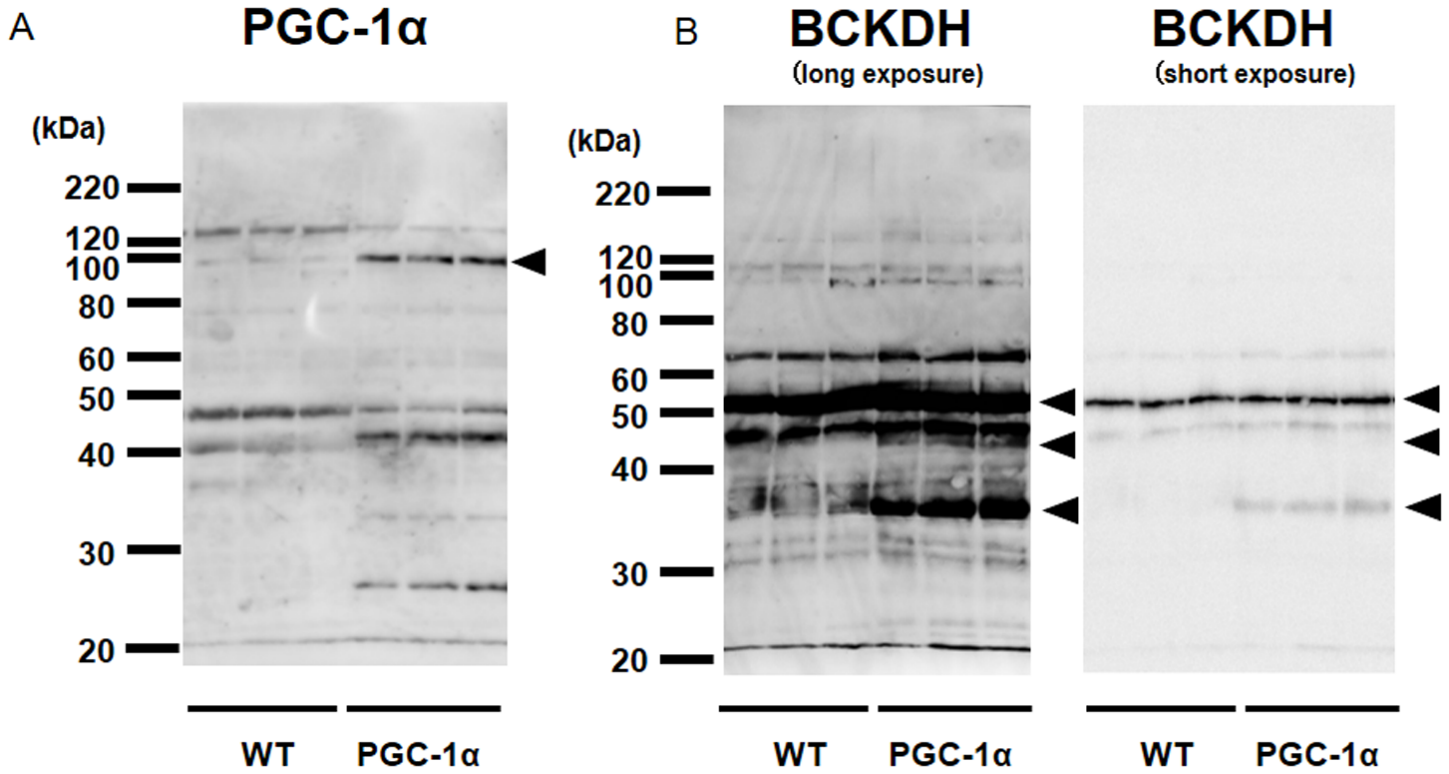
Protein expression of PGC-1α and BCAA metabolic enzymes in skeletal muscle of PGC-1α -Tg mice. Total lysates from skeletal muscle were subjected to SDS-PAGE, followed by Western blot analysis with indicated antibodies. Typical blots are shown. Densitometric analysis was performed on the bands indicated (arrowheads). Molecular size marked was indicated on the left side of blots. Tg and WT mice were sacrificed at 12 weeks of age (N = 3 for WT and N = 3 for Tg mice). In these samples, we confirmed increased mRNA expression of BCAT2 and BCKDH but not BCKDK as observed in Figure 1 (data not shown).

**Figure 4:**
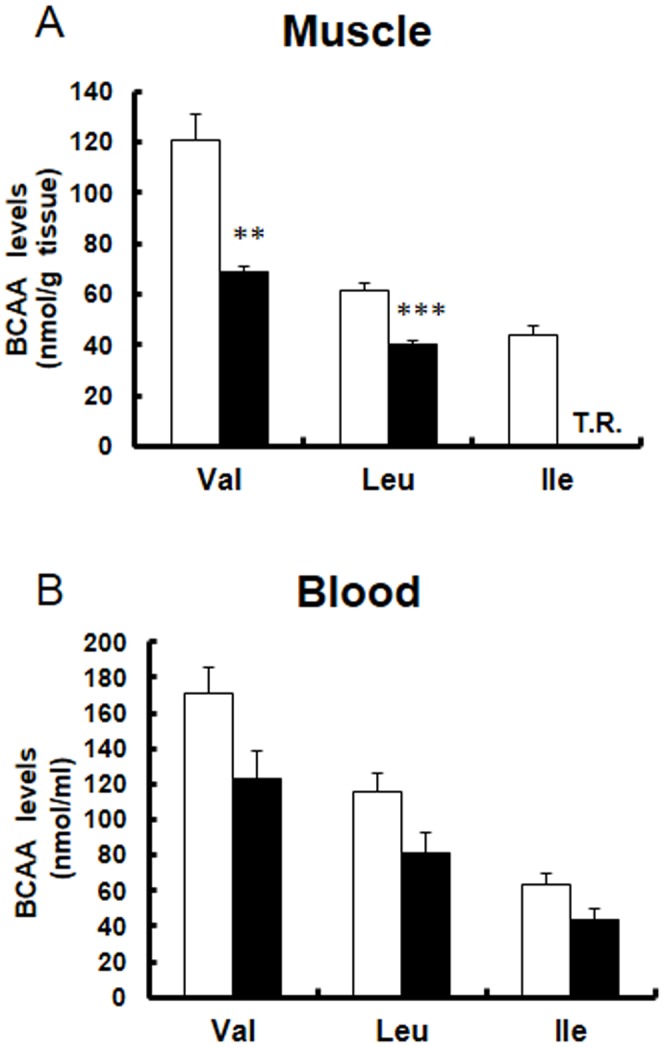
BCAA content in skeletal muscle and blood of PGC-1α Tg mice. Val, Leu, and Ile levels in (A) skeletal muscle and (B) blood. Open columns represent for WT (N = 4) and filled columns represent Tg (N = 4). ***P<0.001, **P<0.01. T.R., trace level.

We examined the gene expression of BCAA metabolic enzymes (BCAT2, BCKDH, BCKDK). RNA was obtained from the skeletal muscle of Tg and WT mice. The expression of BCAT2 (2.0-fold) and BCKDH (3.5-fold) was significantly increased in Tg mice compared with that in WT mice (Fig. 2A, B). Meanwhile, the expression of BCKDK was decreased (Fig. 2C). Subsequently, we examined protein expression of BCAA metabolic enzymes by Western blot analysis. PGC-1α protein (100 kDa) increased 4-fold in Tg mice compared with WT mice (Fig. 3A). In this experiment, we also observed increased 45 kDa and 25 kDa bands, whose physiological significance in currently unclear. Using a BCKDH antibody, we observed the strongest band at 55 kDa (arrowhead), which corresponded to the E2 subunit, and was slightly (1.3-fold) increased in Tg mice. The faint band in WT mice at approximately 45 kDa (arrowhead, long exposure), which probably represents E1α subunits [29], increased in Tg mice significantly (1.5-fold). The band at approximately 35 kDa (arrowhead), which probably represents E1β subunits [29], increased markedly (11-fold). Thus, we observed an increased protein level of BCKDH (Fig. 3B), which is consistent with the increased mRNA level. Next we examined BCAA levels from skeletal muscle in Tg mice and WT mice. Val and Leu levels were significantly decreased in Tg mice compared with that in WT mice (Fig. 4A). Ile was detected in WT mice but observed only at trace levels in Tg mice (Fig. 4A). The level of Glu, a metabolite of BCAA catabolism, was increased, in contrast to the decreased BCAA level. Levels of other amino acids are shown in Table 2. We also measured BCAA levels in blood (plasma). BCAA levels tended to be decreased in blood as observed in skeletal muscle (although not significant, P = 0.067 for Val, P = 0.072 for Leu and P = 0.063 for Ile; Fig. 4B and Table 3). These findings indicate that changes in the expression of BCAA metabolic enzymes are functional, and accompanied by enzyme activation.

**Table 2:** Amino acid content in skeletal muscle of PGC-1α Tg mice.

Amino acid	WT	PGC-1α Tg
Alanine	1768.3	1388.6
Arginine	218.1	446.4*
Asparagine	61.9	53.1
Aspartic acid	163.4	257.3**
Cystine	TR	TR
Glutamic acid	505.9	1237.5***
Glutamine	1341	1557.9
Glycine	3165.9	655.9**
Histidine	104.3	101.3
Isoleucine	44.4	TR
Leucine	61.1	40.3***
Lysine	574	1074.1*
Methionine	49.9	35.7
Phenylalanine	12.4	TR
Proline	169.1	ND
Serine	357.7	186.3*
Threonine	242.6	186.3
Tryptophan	ND	ND
Tyrosine	77	75
Valine	120.8	68.4**

The samples were used as in Figure 4. ***P<0.001, **P<0.01, *P<0.05. TR, trace level. ND, not detected.

**Table 3:** Amino acid content in blood of PGC-1α Tg mice.

Amino acid	WT	PGC-1α Tg
Alanine	381.4	313.9
Arginine	112.3	114.5
Asparagine	45.7	38.9
Aspartic acid	7.7	6.7
Cystine	8.7	5.1
Glutamic acid	42	35.2*
Glutamine	631.4	726
Glycine	306.2	334
Histidine	60.1	58
Isoleucine	63.7	43.6
Leucine	115.3	81.3
Lysine	277.7	256.4
Methionine	56.1	53.1
Phenylalanine	60.5	59.7
Proline	105	96.1
Serine	134.8	125.5
Threonine	117.2	121.6
Tryptophan	41.8	39.7
Tyrosine	99.4	82
Valine	171.1	122.9

The samples were used as in Figure 4.

*P<0.05.

### BCAA metabolism gene expression in C2C12 cells overexpressing PGC-1α

**Figure 5:**
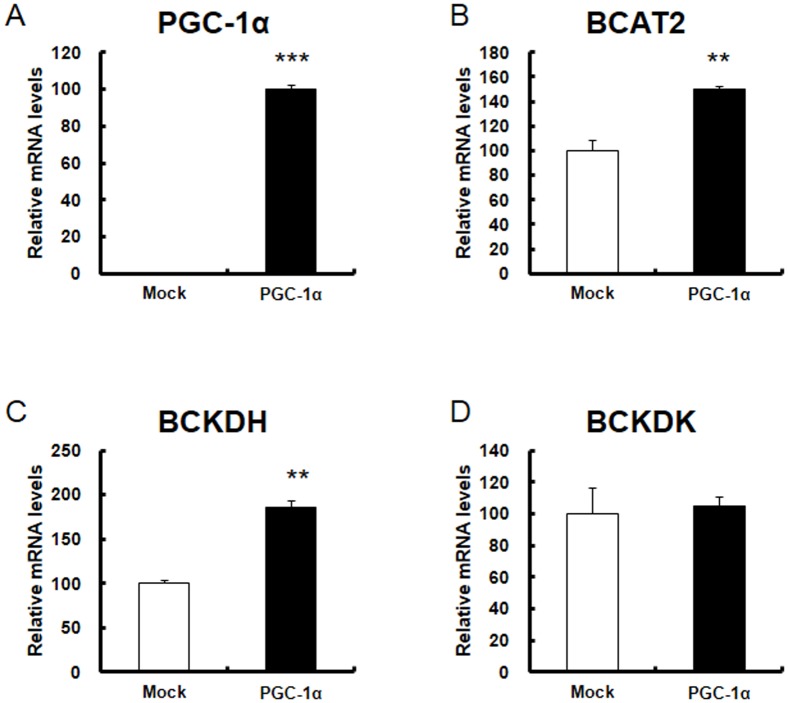
Gene expression of BCAA metabolic enzymes in cultured C2C12 cells overexpressing PGC-1α. Total RNA was isolated from the cells and analyzed by quantitative real-time RT-PCR with primers for A) PGC-1α, B) BCAT2, C) BCKDHa, and D) BCKDK. Open columns represent mock cells (N = 3), and filled columns represent PGC-1α-overexpressed cells (N = 3). Each value represents mean ± SE (N = 3). The relative values are shown (the control is set as 100). For PGC-1α expression, the value was set as 100 in the PGC-1α overexpressed cells. ***P<0.001, **P<0.01.

Next, to examine whether the effect of PGC-1α on increased BCAA metabolism was cell autonomous, we used C2C12 cells, which are ectopically overexpressed PGC-1α by retrovirus, and examined BCAA metabolism gene expression. Gene expression of BCAT2 (1.5-fold) and BCKDH (2-fold) was increased but that of BCKDK was not (Fig. 5), as observed in Tg mice. These data suggest that PGC-1α regulates BCAA catabolic gene expression in a cell autonomous manner. Then, we examined the amino acid levels in the cells. Ile was observed only at a trace level. Val was slightly lower in mock cells than in cells overexpressing PGC-1α (P = 0.162). Leu levels were detectable in mock cells; however, it was detected only at a trace level in cells overexpressing PGC-1α (Table 4). In summary, BCAA levels appeared to be decreased in C2C12 cells overexpressing PGC-1α, suggesting that BCAA catabolism is regulated by PGC-1α in muscle cells.

**Table 4:** Amino acid content in C2C12 cells overexpressing of PGC-1α.

Amino acid	Mock	PGC-1α
Alanine	35.7	62.7*
Arginine	ND	ND
Asparagine	ND	ND
Aspartic acid	44.3	55.3
Cystine	TR	TR
Glutamic acid	116.3	87.1
Glutamine	24.4	20.6
Glycine	67.1	61.3
Histidine	ND	ND
Isoleucine	TR	TR
Leucine	9	TR
Lysine	14.6	15.9
Methionine	ND	ND
Phenylalanine	ND	ND
Proline	ND	ND
Serine	9	9.5
Threonine	27.4	35.2
Tryptophan	ND	ND
Tyrosine	TR	TR
Valine	10.2	8.7

The samples were used as in Figure 5.

*P<0.05. TR, trace level. ND, not detected.

### Changed level of other amino acids caused by PGC-1α overexpression

In addition to BCAA, the levels of other amino acids were also changed. For example, in skeletal muscle of Tg mice, Glu levels were significantly increased (Table 2). During BCAA degradation by BCAT, α-keto glutarate is catabolized to Glu. Thus, increasing Glu levels are consistent with the stimulation of BCAA degradation in muscle. Alternatively, in cells overexpressing PGC-1α, Glu was not increased, but Ala was increased (Table 4). The reason for this may be that BCAT catalyzes amino base transfer from BCAA to pyruvate, thereby producing Ala. However, in Tg mice, the expression of genes involved in glycolysis is markedly decreased [4], resulting in inadequate pyruvate, a product of the glycolysis pathway, for Ala production, or Ala may be moved and used in other tissues in animals.

### How did PGC-1α increase expression of BCAA metabolism enzyme?

PGC-1α Tg mice have muscle, which has a much higher oxidative capacity [4]. BCAT2 and BCKDH are mitochondrial enzymes [30]. Therefore, the increased expression of BCAT2 and BCKDH could be due to an increased number of mitochondria in the muscles of the Tg mice. Alternatively, PGC-1α may activate BCAA metabolism via the coactivation of glucocorticoid receptor (GR) and PPARα. PGC-1α is a transcriptional coactivator of nuclear receptor and other transcriptional factors, some of which have been reported to activate the transcription of the BCAT2 gene. For example, the expression of BCAT2 was decreased in KLF15-KO mice [31], and the rat BCAT2 promoter was activated by KLF15 and GR [32]. Moreover, PPARα activated the BCKDH complex in the liver [22]. In addition, bioinformatics analysis revealed that several nuclear receptors, including PPAR and estrogen receptor-related receptor (ERR), are significantly frequently recruited to the BCAA metabolic pathway genes up-regulated in skeletal muscle of PGC-1α Tg mice (Table 5). These data suggest that PGC-1α activates BCAA metabolism through multiple nuclear receptors.

**Table 5:** Bioinformatics analysis of transcription factors enriched in the BCAA metabolic pathway genes up-regulated in PGC-1α Tg mice.

Transcription factor	P-Value	Target gene
KLF4	2.80E-07	ACAA2;ACADS;BCAT2;BCKDHA;HADHA;MCEE;OXCT1
PPARG	3.68E-07	ACAA2;ACADS;BCAT2;HADHB
EKLF	5.14E-06	ACADS;BCAT2;HADH;HIBADH;OXCT1
ESRRB	1.04E-05	ACADS;DLD;HIBADH;MCEE;OXCT1
PPARD	1.16E-05	HADHA;HADHB;OXCT1
ZFP42	1.22E-05	ACADS;BCAT2;BCKDHA;HADHA;HADHB
WT1	4.22E-04	BCAT2;HADHB;HIBADH;OXCT1
NR0B1	4.49E-04	ACADS;BCAT2;HADHA;HADHB
TET1	6.17E-04	ACAA2;ACADS;HADHA;OXCT1
GATA4	9.09E-04	ACAA2;BCAT2;HADHA;HADHB

List of transcription factors, which are statistically identified as ones that can be recruited to the BCAA metabolic genes, up-regulated in PGC-1α Tg mice. Target genes were previously found in ChIP assay for interacting with indicated transcription factors in the literature [27]. Abbreviations of the transcription factors are as follows, KLF4, Krueppel-like factor 4; PPARG, Constitutive coactivator of peroxisome proliferator-activated receptor gamma (Constitutive coactivator of PPAR-gamma) (Constitutive coactivator of PPARG); EKLF, Krueppel-like factor 1 (Erythroid krueppel-like transcription factor); ESRRB, Steroid hormone receptor ERR2 (Estrogen receptor-like 2) (Estrogen-related receptor beta) (ERR-beta); PPARD, Peroxisome proliferator-activated receptor delta (PPAR-delta); ZFP42, Zinc finger protein 42; WT1, Wilms tumor protein; NR0B1, Nuclear receptor subfamily 0 group B member 1 (Nuclear receptor DAX-1); TET1, Methylcytosine dioxygenase TET1 (EC 1.14.11.n2) (CXXC-type zinc finger protein 6) (Ten-eleven translocation 1 gene protein homolog); GATA4, Transcription factor GATA-4 (GATA-binding factor 4).

## Conclusion

In this study, we investigated whether PGC-1α stimulates BCAA metabolism by increasing the expression of involved enzymes, such as BCAT and BCKDH, using cultured cells and murine skeletal muscle overexpressing PGC-1α. Our data suggest that BCAA degradation is mediated by increased expression of PGC-1α, and the physiological relevance, such as condition of induced expression in PGC-1α i.e., exercise, remain to be analyzed by further experiments.

## Supporting Information

Table S1 **List of genes up-regulated in Tg mice by microarray.** RNA obtained from WT and Tg mice was pooled and used for microarray analysis as described in Methods. Hybridized signals outside the linear range were excluded, and values were normalized by the 75^th^ percentile calculation. Genes with a calculated value less than 100 in Tg mice were deleted. Genes up-regulated more than 2.5 fold in Tg mice were listed. Pooled array data were confirmed by performing quantitative real-time RT-PCR with representative probes and similar increased was observed in all samples, indicating that microarray data represent the expression change in each group (data not shown).
(PDF)
