# Supplementary material for: PGC-1α-Mediated Branched-Chain Amino Acid Metabolism in the Skeletal Muscle
Source: PLoS One. 2014 Mar 17;9(3):e91006. doi: 10.1371/journal.pone.0091006 (PMC3956461; doi:10.1371/journal.pone.0091006)
Supplement: Table S1 — List of genes up-regulated in Tg mice by microarray. RNA obtained from WT and Tg mice was pooled and used for microarray analysis as described in Methods. Hybridized signals outside the linear range were excluded, and values were normalized by the 75th percentile calculation. Genes with a calculated value less than 100 in Tg mice were deleted. Genes up-regulated more than 2.5 fold in Tg mice were listed. Pooled array data were confirmed by performing quantitative real-time RT-PCR with representative probes and similar increased was observed in all samples, indicating that microarray data represent the expression change in each group (data not shown). (PDF) [file pone.0091006.s001.pdf]

Table S1

GeneName	SystematicName	Description	Tg/WT
BC048679	NM_183143	cDNA sequence BC048679 (BC048679)	36.6
Cidea	NM_007702	cell death-inducing DNA fragmentation factor, alpha subunit-like effector A (Cidea)	30.8
Syn2	AK029181	0 day neonate head cDNA, RIKEN full-length enriched library, clone:4831410C22 product:synapsin II, full insert sequence.	14.3
A_51_P502068	A_51_P502068	Unknown	12.3
Tm6sf1	NM_145375	transmembrane 6 superfamily member 1 (Tm6sf1)	11.7
Esrrb	NM_011934	estrogen related receptor, beta (Esrrb)	10.7
Sln	NM_025540	sarcolipin (Sln)	9.0
Mfsd4	NM_172510	major facilitator superfamily domain containing 4 (Mfsd4)	8.9
Paqr4	NM_023824	progestin and adipoQ receptor family member IV (Paqr4)	8.8
Aadacl1	NM_178772	arylacetamide deacetylase-like 1 (Aadacl1)	8.7
Ppif	NM_134084	peptidylprolyl isomerase F (cyclophilin F) (Ppif)	8.6
Rap1gap	BC052065	Rap1 GTPase-activating protein	8.5
Gloxd1	NM_146256	glyoxalase domain containing 1 (Gloxd1)	8.3
Itgb1bp3	NM_027120	integrin beta 1 binding protein 3 (Itgb1bp3)	8.1
1810005K13Rik	XM_885191	PREDICTED: RIKEN cDNA 1810005K13 gene, transcript variant 1	7.9
Ecgf1	NM_138302	endothelial cell growth factor 1 (platelet-derived) (Ecgf1)	7.4
Mt1	NM_013602	metallothionein 1 (Mt1)	7.2
2310076L09Rik	NM_001077348	RIKEN cDNA 2310076L09 gene (2310076L09Rik), transcript variant 2	7.1
Tmem37	NM_019432	transmembrane protein 37 (Tmem37)	7.1
Aspa	NM_023113	aspartoacylase (aminoacylase) 2 (Aspa)	6.8
Slc25a22	NM_026646	solute carrier family 25 (mitochondrial carrier, glutamate), member 22 (Slc25a22)	6.8
P2ry1	NM_008772	purinergic receptor P2Y, G-protein coupled 1 (P2ry1)	6.6
Nrtn	NM_008738	neurturin (Nrtn)	6.3
G0s2	NM_008059	G0/G1 switch gene 2 (G0s2)	6.2
Prdx6-rs1	NM_177256	peroxiredoxin 6, related sequence 1 (Prdx6-rs1)	6.0
Ciapin1	NM_134141	cytokine induced apoptosis inhibitor 1 (Ciapin1)	5.8
Ccl8	NM_021443	chemokine (C-C motif) ligand 8 (Ccl8)	5.8
Gmnn	NM_020567	geminin (Gmnn)	5.8
Prdx6	NM_007453	peroxiredoxin 6 (Prdx6)	5.7
Car2	NM_009801	carbonic anhydrase 2 (Car2)	5.7
Ung	NM_001040691	uracil DNA glycosylase (Ung), transcript variant 1	5.7
Mid1ip1	NM_026524	Mid1 interacting protein 1 (gastrulation specific G12-like (zebrafish)) (Mid1ip1)	5.6
Tmem25	NM_027865	transmembrane protein 25 (Tmem25)	5.4
NAP053239-1	NAP053239-1	Unknown	5.2
BC022224	NM_177564	cDNA sequence BC022224 (BC022224)	5.1
Pdss1	NM_019501	prenyl (solanesyl) diphosphate synthase, subunit 1 (Pdss1)	5.1
Iqcg	NM_178378	IQ motif containing G (Iqcg)	5.1
Gadd45g	NM_011817	growth arrest and DNA-damage-inducible 45 gamma (Gadd45g)	5.0
Ldhb	NM_008492	lactate dehydrogenase B (Ldhb)	5.0
1300010F03Rik	BC019143	RIKEN cDNA 1300010F03 gene	5.0
Prdx6-rs2	ENSMUST00000519	PREDICTED: peroxiredoxin 6, related sequence 2 (Prdx6-rs2)	4.9
Mrm1	NM_145433	mitochondrial rRNA methyltransferase 1 homolog (S. cerevisiae) (Mrm1)	4.9
Cish	NM_009895	cytokine inducible SH2-containing protein (Cish)	4.8
Slc41a1	NM_173865	solute carrier family 41, member 1 (Slc41a1)	4.8
NAP028712-1	NAP028712-1	Unknown	4.7
Snn	NM_009223	stannin (Snn)	4.7
Nudt8	NM_025529	nudix (nucleoside diphosphate linked moiety X)-type motif 8 (Nudt8)	4.6
Msrb2	NM_029619	methionine sulfoxide reductase B2 (Msrb2)	4.6
Impa2	NM_053261	inositol (myo)-1(or 4)-monophosphatase 2 (Impa2)	4.6
Abcb9	NM_019875	ATP-binding cassette, sub-family B (MDR/TAP), member 9 (Abcb9)	4.6
Acaa2	NM_177470	acetyl-Coenzyme A acyltransferase 2 (mitochondrial 3-oxoacyl-Coenzyme A	4.5
Hhat	NM_144881	hedgehog acyltransferase (Hhat)	4.5
Pnpla2	NM_025802	patatin-like phospholipase domain containing 2 (Pnpla2)	4.5
Car7	NM_053070	carbonic anhydrase 7 (Car7)	4.5
Coq6	NM_172582	coenzyme Q6 homolog (yeast) (Coq6)	4.5
9930105H17Rik	AK037062	adult female vagina cDNA, RIKEN full-length enriched library, clone:9930105H17 product:weakly similar to ENV POLYPROTEIN PRECURSOR	4.4
Mdh1	NM_008618	malate dehydrogenase 1, NAD (soluble) (Mdh1)	4.4
Coq7	NM_009940	demethyl-Q 7 (Coq7)	4.4
Ckmt2	NM_198415	creatine kinase, mitochondrial 2 (Ckmt2)	4.4
Vegfb	NM_011697	vascular endothelial growth factor B (Vegfb)	4.4
Pctk3	NM_008795	PCTAIRE-motif protein kinase 3 (Pctk3)	4.4
Acsl1	NM_007981	acyl-CoA synthetase long-chain family member 1 (Acsl1)	4.4
1700022A21Rik	XM_131911	PREDICTED: RIKEN cDNA 1700022A21 gene (1700022A21Rik)	4.4
1100001H23Rik	NM_025806	RIKEN cDNA 1100001H23 gene (1100001H23Rik)	4.4
Got1	NM_010324	glutamate oxaloacetate transaminase 1, soluble (Got1)	4.4
Casq2	NM_009814	calsequestrin 2 (Casq2)	4.3
Fndc5	NM_027402	fibronectin type III domain containing 5 (Fndc5)	4.3
Nlrp2	NM_177690	NLR family, pyrin domain containing 2 (Nlrp2)	4.2
6720475J19Rik	XM_983620	PREDICTED: RIKEN cDNA 6720475J19 gene (6720475J19Rik)	4.2
Acadvl	NM_017366	acyl-Coenzyme A dehydrogenase, very long chain (Acadvl)	4.2
Dcakd	NM_026551	dephospho-CoA kinase domain containing (Dcakd)	4.2
Prdx5	NM_012021	peroxiredoxin 5 (Prdx5)	4.2
Sirt5	NM_178848	sirtuin 5 (silent mating type information regulation 2 homolog) 5 (S. cerevisiae)	4.1
AV124335	AV124335	AV124335 AV124335 C57BL/6J 11-day embryo cDNA clone 2700017N2	4.1
Phyh	NM_010726	phytanoyl-CoA hydroxylase (Phyh)	4.1

S100a1	NM_011309	S100 calcium binding protein A1 (S100a1)	4.1
Got2	NM_010325	glutamate oxaloacetate transaminase 2, mitochondrial (Got2)	4.1
Cabc1	NM_023341	chaperone, ABC1 activity of bc1 complex like (S. pombe) (Cabc1)	4.0
Ccbl2	NM_173763	cysteine conjugate-beta lyase 2 (Ccbl2)	4.0
Hadha	NM_178878	hydroxyacyl-Coenzyme A dehydrogenase/3-ketoacyl-Coenzyme A thiolase/enoyl-Coenzyme A hydratase (trifunctional protein), alpha subunit (Hadha)	4.0
1500026H17Rik	AK048993	0 day neonate cerebellum cDNA, RIKEN full-length enriched library, clone:C230089J09 product:weakly similar to GAG POLYPROTEIN	4.0
Slc25a20	NM_020520	solute carrier family 25 (mitochondrial carnitine/acylcarnitine translocase), member 20 (Slc25a20)	4.0
Atad3a	NM_179203	ATPase family, AAA domain containing 3A (Atad3a)	4.0
Coq5	NM_026504	coenzyme Q5 homolog, methyltransferase (yeast) (Coq5)	3.9
AW146242	NM_146168	expressed sequence AW146242	3.9
Mrpl47	NM_029017	mitochondrial ribosomal protein L47 (Mrpl47)	3.9
D16H22S680E	NM_138583	DNA segment, Chr 16, human D22S680E, expressed (D16H22S680E)	3.9
Adck1	NM_028105	aarF domain containing kinase 1 (Adck1)	3.9
Atp5g1	NM_007506	ATP synthase, H+ transporting, mitochondrial F0 complex, subunit c (subunit 9), isoform 1 (Atp5g1)	3.9
Mt2	NM_008630	metallothionein 2 (Mt2)	3.8
1700020C11Rik	NM_026443	RIKEN cDNA 1700020C11 gene (1700020C11Rik)	3.8
8430408G22Rik	NM_145980	RIKEN cDNA 8430408G22 gene (8430408G22Rik)	3.8
Qrsl1	BC070459	glutaminyl-tRNA synthase (glutamine-hydrolyzing)-like 1	3.8
Nme1	NM_008704	expressed in non-metastatic cells 1, protein (Nme1)	3.8
Dlst	NM_030225	dihydrolipoamide S-succinyltransferase (E2 component of 2-oxo-glutarate complex)	3.8
Nudt7	NM_024437	nudix (nucleoside diphosphate linked moiety X)-type motif 7 (Nudt7), transcript	3.8
Fdxr	NM_007997	ferredoxin reductase (Fdxr)	3.8
Ethe1	NM_023154	ethylmalonic encephalopathy 1 (Ethe1)	3.7
NAP046356-1	NAP046356-1	Unknown	3.7
Chpt1	NM_144807	choline phosphotransferase 1 (Chpt1)	3.7
Ndufs8	NM_144870	NADH dehydrogenase (ubiquinone) Fe-S protein 8 (Ndufs8)	3.7
Decr1	NM_026172	2,4-dienoyl CoA reductase 1, mitochondrial (Decr1)	3.7
Acadl	NM_007381	acyl-Coenzyme A dehydrogenase, long-chain (Acadl)	3.6
Mtx2	NM_016804	metaxin 2 (Mtx2)	3.6
Ppara	NM_011144	peroxisome proliferator activated receptor alpha (Ppara)	3.6
Mrpl45	NM_025927	mitochondrial ribosomal protein L45 (Mrpl45)	3.6
Dscr1l1	NM_207649	Down syndrome critical region gene 1-like 1 (Dscr1l1), transcript variant 1	3.6
Retsat	NM_026159	retinol saturase (all trans retinol 13,14 reductase) (Retsat)	3.6
2410003P15Rik	NM_018888	RIKEN cDNA 2410003P15 gene (2410003P15Rik)	3.6
Il10rb	NM_008349	interleukin 10 receptor, beta (Il10rb)	3.6
Gpt1	NM_182805	glutamic pyruvic transaminase 1, soluble (Gpt1)	3.6
Mrpl19	NM_026490	mitochondrial ribosomal protein L19 (Mrpl19)	3.5
1810049H13Rik	NM_025560	RIKEN cDNA 1810049H13 gene (1810049H13Rik)	3.5
Akap1	NM_001042541	A kinase (PRKA) anchor protein 1 (Akap1), transcript variant 2	3.5
Myh1	NM_030679	myosin, heavy polypeptide 1, skeletal muscle, adult (Myh1)	3.5
Gdpd1	NM_025638	glycerophosphodiester phosphodiesterase domain containing 1 (Gdpd1)	3.5
ENSMUST0000008706	ENSMUST00000087C	similar to solute carrier family 25, member 5 (LOC673916)	3.5
Bckdha	NM_007533	branched chain ketoacid dehydrogenase E1, alpha polypeptide (Bckdha)	3.5
Dlat	NM_145614	dihydrolipoamide S-acetyltransferase (E2 component of pyruvate dehydrogenase complex) (Dlat)	3.5
2310061C15Rik	NM_026844	RIKEN cDNA 2310061C15 gene (2310061C15Rik)	3.5
Zfpm1	NM_009569	zinc finger protein, multitype 1 (Zfpm1)	3.5
Fabp3	NM_010174	fatty acid binding protein 3, muscle and heart (Fabp3)	3.5
Lace1	NM_145743	lactation elevated 1 (Lace1)	3.5
Endog	NM_007931	endonuclease G (Endog)	3.4
1110019J04Rik	XM_898905	PREDICTED: RIKEN cDNA 1110019J04 gene, transcript variant 2	3.4
Hba-a1	NM_008218	hemoglobin alpha, adult chain 1 (Hba-a1)	3.4
Ccdc90a	AK018089	11 days embryo head cDNA, RIKEN full-length enriched library, clone:6230416A05	3.4
9530058B02Rik	NM_026633	RIKEN cDNA 9530058B02 gene (9530058B02Rik)	3.4
1810011O10Rik	NM_026931	RIKEN cDNA 1810011O10 gene (1810011O10Rik)	3.4
2410005O16Rik	NM_025476	RIKEN cDNA 2410005O16 gene (2410005O16Rik)	3.4
Rbpms2	NM_028030	RNA binding protein with multiple splicing 2 (Rbpms2)	3.4
Slc25a29	NM_181328	solute carrier family 25 (mitochondrial carrier, palmitoylcarnitine transporter), member 29 (Slc25a29)	3.4
Slc25a5	NM_007451	solute carrier family 25 (mitochondrial carrier, adenine nucleotide translocator), member 5 (Slc25a5)	3.4
Rbp7	NM_022020	retinol binding protein 7, cellular (Rbp7)	3.4
Hadh	NM_008212	hydroxyacyl-Coenzyme A dehydrogenase (Hadh)	3.4
TC1703837	TC1703837	CNBP_MOUSE (P53996) Cellular nucleic acid-binding protein (CNBP) (Zinc finger protein 9)	3.4
Supv3l1	NM_181423	suppressor of var1, 3-like 1 (S. cerevisiae) (Supv3l1)	3.4
Brp44l	NM_018819	brain protein 44-like (Brp44l)	3.4
Prdx2	NM_011563	peroxiredoxin 2 (Prdx2)	3.3
Ech1	NM_016772	enoyl coenzyme A hydratase 1, peroxisomal (Ech1)	3.3
Dgat2	NM_026384	diacylglycerol O-acyltransferase 2 (Dgat2)	3.3
Sdhb	NM_023374	succinate dehydrogenase complex, subunit B, iron sulfur (Ip) (Sdhb)	3.3
Yars2	NM_198246	tyrosyl-tRNA synthetase 2 (mitochondrial) (Yars2)	3.3
Alad	NM_008525	aminolevulinate, delta-, dehydratase (Alad)	3.3
Scfd2	AK143615	6 days neonate spleen cDNA, RIKEN full-length enriched library, clone:F430003B18 product:sec1 family domain containing 2	3.3

Kcnn1	NM_032397	potassium intermediate/small conductance calcium-activated channel, subfamily N, member 1 (Kcnn1)	3.3
NAP113002-1	NAP113002-1	Unknown	3.3
Gfm1	AK018125	adult male medulla oblongata cDNA, RIKEN full-length enriched library, clone:6330405J24 product:G elongation factor	3.3
Ddt	NM_010027	D-dopachrome tautomerase (Ddt)	3.3
Tnfaip2	NM_009396	tumor necrosis factor, alpha-induced protein 2 (Tnfaip2)	3.3
5133401N09Rik	NM_198004	RIKEN cDNA 5133401N09 gene (5133401N09Rik), transcript variant 1	3.2
Uqcrc1	NM_025407	ubiquinol-cytochrome c reductase core protein 1 (Uqcrc1)	3.2
2610029I01Rik	AK154853	NOD-derived CD11c +ve dendritic cells cDNA, RIKEN full-length enriched library, clone:F630109L08 product:hypothetical Rhodanese-like/Rhodanese domain profile/Thiosulfate sulfurtransferase containing protein	3.2
Glrx5	NM_028419	glutaredoxin 5 homolog (S. cerevisiae) (Glrx5)	3.2
Atp1b1	NM_009721	ATPase, Na+/K+ transporting, beta 1 polypeptide (Atp1b1)	3.2
Cd36	NM_007643	CD36 antigen (Cd36)	3.2
Sirt3	NM_022433	sirtuin 3 (silent mating type information regulation 2, homolog) 3 (S. cerevisiae)	3.1
Cd300lg	NM_027987	CD300 antigen like family member G (Cd300lg)	3.1
Cpt2	NM_009949	carnitine palmitoyltransferase 2 (Cpt2)	3.1
Tcn2	NM_015749	transcobalamin 2 (Tcn2)	3.1
Hsdl2	NM_024255	hydroxysteroid dehydrogenase like 2 (Hsdl2)	3.1
Slc16a1	NM_009196	solute carrier family 16 (monocarboxylic acid transporters), member 1 (Slc16a1)	3.1
Mb	NM_013593	myoglobin (Mb)	3.1
Ldhd	NM_027570	lactate dehydrogenase D (Ldhd)	3.1
Smtnl1	NM_024230	smoothelin-like 1 (Smtnl1)	3.1
Vegfa	NM_001025250	vascular endothelial growth factor A (Vegfa), transcript variant 1	3.1
D430028G21Rik	NM_144888	RIKEN cDNA D430028G21 gene (D430028G21Rik)	3.1
Hmgb3	NM_008253	high mobility group box 3 (Hmgb3)	3.1
Alas1	NM_020559	aminolevulinic acid synthase 1 (Alas1)	3.1
Mrpl14	NM_026732	mitochondrial ribosomal protein L14 (Mrpl14)	3.0
Hbb-b1	NM_008220	hemoglobin, beta adult major chain (Hbb-b1)	3.0
Cyb561d1	BC043107	cytochrome b-561 domain containing 1	3.0
Atp5b	NM_016774	ATP synthase, H+ transporting mitochondrial F1 complex, beta subunit (Atp5b), nuclear gene encoding mitochondrial protein	3.0
Mustn1	NM_181390	musculoskeletal, embryonic nuclear protein 1 (Mustn1)	3.0
BC021611	NM_144932	cDNA sequence BC021611 (BC021611)	3.0
Phb	NM_008831	prohibitin (Phb)	3.0
Timm10	NM_013899	translocase of inner mitochondrial membrane 10 homolog (yeast) (Timm10)	3.0
Tspan12	NM_173007	tetraspanin 12 (Tspan12)	3.0
Osbpl1a	BC076637	oxysterol binding protein-like 1A	3.0
AV104666	AV104666	liver C57BL/6J 13-day embryo cDNA clone 2510008K06	3.0
Adamtsl5	BC034843	ADAMTS-like 5	3.0
Dld	NM_007861	dihydrolipoamide dehydrogenase (Dld)	3.0
ORF28	BC005604	open reading frame 28	3.0
Alas2	NM_009653	aminolevulinic acid synthase 2, erythroid (Alas2)	3.0
Mterfd3	NM_028832	MTERF domain containing 3 (Mterfd3)	3.0
Aldh5a1	NM_172532	aldhehyde dehydrogenase family 5, subfamily A1 (Aldh5a1)	3.0
Mrpl3	NM_053159	mitochondrial ribosomal protein L3 (Mrpl3)	3.0
Abca2	NM_007379	ATP-binding cassette, sub-family A (ABC1), member 2 (Abca2)	3.0
Pmm1	NM_013872	phosphomannomutase 1 (Pmm1)	2.9
Naprt1	NM_172607	nicotinate phosphoribosyltransferase domain containing 1 (Naprt1)	2.9
Rbck1	NM_019705	RanBP-type and C3HC4-type zinc finger containing 1 (Rbck1)	2.9
LOC434179	NM_001008427	hypothetical LOC434179 (LOC434179)	2.9
Fdx1	NM_007996	ferredoxin 1 (Fdx1)	2.9
Etfdh	NM_025794	electron transferring flavoprotein, dehydrogenase (Etfdh)	2.9
Gsto1	NM_010362	glutathione S-transferase omega 1 (Gsto1)	2.9
2310004L02Rik	NM_025504	RIKEN cDNA 2310004L02 gene (2310004L02Rik)	2.9
Oxct1	NM_024188	3-oxoacid CoA transferase 1 (Oxct1)	2.9
Oplah	NM_153122	5-oxoprolinase (ATP-hydrolysing) (Oplah)	2.9
Esrra	NM_007953	estrogen related receptor, alpha (Esrra)	2.9
Blnk	NM_008528	B-cell linker (Blnk)	2.9
Lgals3	NM_010705	lectin, galactose binding, soluble 3 (Lgals3)	2.9
Sod2	NM_013671	superoxide dismutase 2, mitochondrial (Sod2)	2.9
Ctsf	NM_019861	cathepsin F (Ctsf)	2.9
Srxn1	NM_029688	sulfiredoxin 1 homolog (S. cerevisiae) (Srxn1)	2.9
Ncapd2	NM_146171	non-SMC condensin I complex, subunit D2 (Ncapd2)	2.9
Ndufa10	NM_024197	NADH dehydrogenase (ubiquinone) 1 alpha subcomplex 10 (Ndufa10)	2.9
Brp44	NM_027430	brain protein 44 (Brp44)	2.9
Pscd1	NM_011180	pleckstrin homology, Sec7 and coiled-coil domains 1 (Pscd1)	2.8
Rasip1	NM_028544	Ras interacting protein 1 (Rasip1)	2.8
Idh2	NM_173011	isocitrate dehydrogenase 2 (NADP+), mitochondrial (Idh2)	2.8
Ucp3	NM_009464	uncoupling protein 3 (mitochondrial, proton carrier) (Ucp3)	2.8
Id3	NM_008321	inhibitor of DNA binding 3 (Id3)	2.8
Mrpl46	NM_023331	mitochondrial ribosomal protein L46 (Mrpl46)	2.8
Timp4	NM_080639	tissue inhibitor of metalloproteinase 4 (Timp4)	2.8
Ephx2	NM_007940	epoxide hydrolase 2, cytoplasmic (Ephx2)	2.8
Pdhx	NM_175094	pyruvate dehydrogenase complex, component X (Pdhx)	2.8
Slc25a39	NM_026542	solute carrier family 25, member 39 (Slc25a39)	2.8
Tufm	NM_172745	Tu translation elongation factor, mitochondrial (Tufm)	2.8
Rmnd1	NM_025343	required for meiotic nuclear division 1 homolog (S. cerevisiae) (Rmnd1)	2.8
CB588406	CB588406	AGENCOURT_12600125 NIH_MGC_136	2.8

Ptcd2	NM_026873	pentatricopeptide repeat domain 2 (Ptcd2)	2.8
Nt5m	NM_134029	5',3'-nucleotidase, mitochondrial (Nt5m)	2.8
Mrpl4	NM_023167	mitochondrial ribosomal protein L4 (Mrpl4)	2.8
9530028C05	AK035387	adult male urinary bladder cDNA, RIKEN full-length enriched library, clone:9530028C05 product:similar to HISTOCOMPATIBILITY 2, CLASS II	2.8
Ttc19	NM_028360	tetratricopeptide repeat domain 19 (Ttc19), transcript variant 1	2.8
Chrna1	NM_007389	cholinergic receptor, nicotinic, alpha polypeptide 1 (muscle) (Chrna1)	2.8
Dci	NM_010023	dodecenoyl-Coenzyme A delta isomerase (3,2 trans-enoyl-Coenyme A isomerase)	2.8
Usp2	NM_198092	ubiquitin specific peptidase 2 (Usp2), transcript variant 3	2.8
Ndufa11	BC059729	NADH dehydrogenase (ubiquinone) 1 alpha subcomplex 11	2.8
D930015E06Rik	ENSMUST000000523	RIKEN cDNA D930015E06 gene	2.8
Cox6b1	NM_025628	cytochrome c oxidase, subunit VIb polypeptide 1 (Cox6b1)	2.8
Ndufaf1	NM_027175	NADH dehydrogenase (ubiquinone) 1 alpha subcomplex, assembly factor 1	2.8
Grpel2	NM_021296	GrpE-like 2, mitochondrial (Grpel2), nuclear gene encoding mitochondrial protein	2.8
Mrpl37	NM_025500	mitochondrial ribosomal protein L37 (Mrpl37)	2.8
Mpeg1	XM_129176	PREDICTED: macrophage expressed gene 1, transcript variant 1 (Mpeg1)	2.8
Notch4	NM_010929	Notch gene homolog 4 (Drosophila) (Notch4)	2.7
Mrps35	NM_145573	mitochondrial ribosomal protein S35 (Mrps35)	2.7
Olfr1497	NM_146741	olfactory receptor 1497 (Olfr1497)	2.7
Pacsin2	NM_011862	protein kinase C and casein kinase substrate in neurons 2 (Pacsin2)	2.7
Acads	NM_007383	acyl-Coenzyme A dehydrogenase, short chain (Acads)	2.7
Cyc1	NM_025567	cytochrome c-1 (Cyc1)	2.7
Aco2	NM_080633	aconitase 2, mitochondrial (Aco2)	2.7
Anks1	NM_181413	ankyrin repeat and SAM domain containing 1 (Anks1)	2.7
1300001I01Rik	AK154925	NOD-derived CD11c +ve dendritic cells cDNA, RIKEN full-length enriched library, clone:F630111I05 product:Putative eukaryotic translation initiation factor 3 subunit	2.7
Hadhb	NM_145558	hydroxyacyl-Coenzyme A dehydrogenase/3-ketoacyl-Coenzyme A thiolase/enoyl-Coenzyme A hydratase (trifunctional protein), beta subunit (Hadhb)	2.7
Atp5d	NM_025313	ATP synthase, H+ transporting, mitochondrial F1 complex, delta subunit (Atp5d)	2.7
Mrps27	NM_173757	mitochondrial ribosomal protein S27 (Mrps27)	2.7
1810022C23Rik	NM_026947	RIKEN cDNA 1810022C23 gene (1810022C23Rik)	2.7
Serpinb1a	NM_025429	serine (or cysteine) peptidase inhibitor, clade B, member 1a (Serpinb1a)	2.7
Ttl	NM_027192	tubulin tyrosine ligase (Ttl)	2.7
ENSMUST0000009772	ENSMUST000000977	CASP8 and FADD-like apoptosis regulator	2.7
Ndor1	NM_178239	NADPH dependent diflavin oxidoreductase 1 (Ndor1)	2.7
Sema7a	NM_011352	sema domain, immunoglobulin domain (Ig), and GPI membrane anchor, (semaphorin) 7A (Sema7a)	2.7
Mcee	NM_028626	methylmalonyl CoA epimerase (Mcee)	2.7
Gbe1	NM_028803	glucan (1,4-alpha-), branching enzyme 1 (Gbe1)	2.7
Mif	NM_010798	macrophage migration inhibitory factor (Mif)	2.6
Shank3	AJ245904	mRNA for Shank3b protein (shank3 gene)	2.6
Junb	NM_008416	Jun-B oncogene (Junb)	2.6
Hbld1	BC022589	HESB like domain containing 1	2.6
Fbxl15	NM_133694	F-box and leucine-rich repeat protein 15 (Fbxl15)	2.6
Npal3	NM_028995	NIPA-like domain containing 3 (Npal3)	2.6
Mrpl41	NM_001031808	mitochondrial ribosomal protein L41 (Mrpl41)	2.6
Tbrg4	NM_134011	transforming growth factor beta regulated gene 4 (Tbrg4)	2.6
D330001F17Rik	AK090131	colon RCB-0549 Cle-H3 cDNA, RIKEN full-length enriched library, clone:G431002O08 product:weakly similar to KIAA1833 PROTEIN	2.6
Wipi1	NM_145940	WD repeat domain, phosphoinositide interacting 1 (Wipi1)	2.6
Cs	NM_026444	citrate synthase (Cs)	2.6
Ogdh	NM_010956	oxoglutarate dehydrogenase (lipoamide) (Ogdh)	2.6
Pdxk	AK009645	adult male tongue cDNA, RIKEN full-length enriched library, clone:2310036D04	2.6
Mrpl12	NM_027204	mitochondrial ribosomal protein L12 (Mrpl12)	2.6
Tie1	NM_011587	tyrosine kinase receptor 1 (Tie1)	2.6
TC1652077	TC1652077	Q8XIF6_CLOPE (Q8XIF6) Probable glycoprotein endopeptidase, partial (6%)	2.6
Csl	NM_027945	citrate synthase like (Csl)	2.6
Ndufs4	NM_010887	NADH dehydrogenase (ubiquinone) Fe-S protein 4 (Ndufs4)	2.6
Ppp1r14b	NM_008889	protein phosphatase 1, regulatory (inhibitor) subunit 14B (Ppp1r14b)	2.6
1810015C04Rik	NM_025459	RIKEN cDNA 1810015C04 gene (1810015C04Rik), transcript variant 2	2.6
Ndufv2	NM_028388	NADH dehydrogenase (ubiquinone) flavoprotein 2 (Ndufv2)	2.6
Cox6a1	NM_007748	cytochrome c oxidase, subunit VI a, polypeptide 1 (Cox6a1)	2.6
2400006H24Rik	NM_021329	RIKEN cDNA 2400006H24 gene (2400006H24Rik)	2.6
Hibadh	NM_145567	3-hydroxyisobutyrate dehydrogenase (Hibadh)	2.6
Evc	NM_021292	Ellis van Creveld gene homolog (human) (Evc)	2.6
Slc25a3	NM_133668	solute carrier family 25 (mitochondrial carrier, phosphate carrier), member 3	2.6
Atp5f1	BC049640	ATP synthase, H+ transporting, mitochondrial F0 complex, subunit b, isoform 1	2.6
Entpd4	NM_026174	ectonucleoside triphosphate diphosphohydrolase 4 (Entpd4)	2.5
Dirc2	NM_153550	disrupted in renal carcinoma 2 (human) (Dirc2)	2.5
Gpr56	NM_018882	G protein-coupled receptor 56 (Gpr56)	2.5
Mrpl18	NM_026310	mitochondrial ribosomal protein L18 (Mrpl18)	2.5
Glrx	NM_053108	glutaredoxin (Glrx)	2.5
Acss2	NM_019811	acyl-CoA synthetase short-chain family member 2 (Acss2)	2.5
Etfb	NM_026695	electron transferring flavoprotein, beta polypeptide (Etfb)	2.5
Sdha	NM_023281	succinate dehydrogenase complex, subunit A, flavoprotein (Fp) (Sdha)	2.5
Timm8a1	NM_013898	translocase of inner mitochondrial membrane 8 homolog a1 (yeast) (Timm8a1)	2.5
Ndufa4	NM_010886	NADH dehydrogenase (ubiquinone) 1 alpha subcomplex, 4 (Ndufa4)	2.5
Rtn4ip1	NM_130892	reticulon 4 interacting protein 1 (Rtn4ip1)	2.5
2810410L24Rik	NM_175239	RIKEN cDNA 2810410L24 gene (2810410L24Rik)	2.5
Klc4	NM_029091	kinesin light chain 4 (Klc4)	2.5

Bcat2	NM_009737	branched chain aminotransferase 2, mitochondrial (Bcat2)	2.5
Tmem53	NM_026837	transmembrane protein 53 (Tmem53)	2.5
NAP061630-1	NAP061630-1	Unknown	2.5
Mfhas1	BC082308	malignant fibrous histiocytoma amplified sequence 1	2.5
Zfp691	NM_183140	zinc finger protein 691 (Zfp691)	2.5
Cox4i1	NM_009941	cytochrome c oxidase subunit IV isoform 1 (Cox4i1)	2.5
Ndufab1	NM_028177	NADH dehydrogenase (ubiquinone) 1, alpha/beta subcomplex, 1 (Ndufab1)	2.5
Gpihbp1	NM_026730	GPI-anchored HDL-binding protein 1 (Gpihbp1)	2.5
Ndufa6	NM_025987	NADH dehydrogenase (ubiquinone) 1 alpha subcomplex, 6 (B14) (Ndufa6)	2.5
Tpst2	NM_009419	protein-tyrosine sulfotransferase 2 (Tpst2)	2.5
Itga6	BC024571	integrin alpha 6	2.5
Hccs	NM_008222	holocytochrome c synthetase (Hccs)	2.5
Inppl1	NM_010567	inositol polyphosphate phosphatase-like 1 (Inppl1)	2.5
Rnf5	NM_019403	ring finger protein 5 (Rnf5)	2.5
Ndufs3	NM_026688	NADH dehydrogenase (ubiquinone) Fe-S protein 3 (Ndufs3)	2.5
Mrpl34	NM_053162	mitochondrial ribosomal protein L34 (Mrpl34)	2.5
Scarb1	NM_016741	scavenger receptor class B, member 1 (Scarb1)	2.5
Cox8a	NM_007750	cytochrome c oxidase, subunit VIIIa (Cox8a)	2.5
Pdhb	NM_024221	pyruvate dehydrogenase (lipoamide) beta (Pdhb)	2.5
D10Jhu81e	NM_138601	DNA segment, Chr 10, Johns Hopkins University 81 expressed (D10Jhu81e)	2.5

Table S1 List of genes up-regulated in Tg mice by microarray

RNA obtained from WT and Tg mice was pooled and used for microarray analysis as described in Methods. Hybridized signals outside the linear range were excluded, and values were normalized by the 75th percentile calculation. Genes with a calculated value less than 100 in Tg mice were deleted. Genes up-regulated more than 2.5 fold in Tg mice were listed. Pooled array data were confirmed by performing quantitative real-time RT-PCR with representative probes and similar increased was observed in all samples, indicating that microarray data represent the expression change in each group (data not shown).
